# Antigenic and genetic characterization of influenza viruses isolated in Mozambique during the 2015 season

**DOI:** 10.1371/journal.pone.0201248

**Published:** 2018-07-26

**Authors:** Almiro Tivane, Rodney Daniels, Neuza Nguenha, Loira Machalele, Afonso Nacoto, Mirela Pale, Edirsse Mateonane, Sandra Mavale, Josina Chilundo, Délcio Muteto, Judite Salência, Félix Albati, Eduardo Gudo, Tufária Mussá, John McCauley

**Affiliations:** 1 Department of Technologic Platforms, Instituto Nacional de Saúde, Ministry of Health, Maputo, Mozambique; 2 Francis Crick Institute, Worldwide Influenza Centre, London, United Kingdom; 3 Pediatric Department, Maputo Central Hospital, Maputo, Mozambique; 4 Department of Microbiology, Faculty of Medicine, Eduardo Mondlane University, Maputo, Mozambique; Sun Yat-Sen University, CHINA

## Abstract

**Background:**

Due to the high rate of antigenic variation of influenza virus, seasonal characterization of the virus is crucial to assess and monitor the emergence of new pathogenic variants and hence formulate effective control measures. However, no study has yet been conducted in Mozambique to assess genetic, antigenic and antiviral susceptibility profile of influenza virus.

**Methods:**

A subset of samples (n = 20) from influenza positive children detected in two hospitals in Maputo city during 2015 season as part of the implementation of influenza surveillance system, were selected. The following assays were performed on these samples: antigenic characterization by hemagglutination inhibition assay, genetic characterization by Sanger sequencing of hemagglutinin (HA) and neuraminidase (NA) and susceptibility to oseltamivir and zanamivir (NA inhibitors) by enzymatic assay.

**Results:**

The A(H1N1)pdm09 subtype viruses remained closely related antigenically and genetically to the 2016 vaccine virus A/California/7/2009 and other widely distributed viruses belonging to genetic group 6B. The majority of influenza A(H3N2) viruses studied were antigenically similar to the 2016–2017 vaccine virus, A/Hong Kong/4801/2014, and their HA and NA gene sequences fell into genetic subclade 3C.2a being closely related to viruses circulating in southern Africa. The influenza B viruses were antigenically similar to the 2016 season vaccine virus and HA sequences of all three fell into the B/Yamagata-lineage, clade 3, but contained NA genes of the B/Victoria-lineage. All tested viruses were sensitive to oseltamivir and zanamivir.

**Conclusion:**

Overall, all Mozambican influenza A and B viruses were most closely related to Southern African viruses and all were sensitive to oseltamivir and zanamivir. These findings suggest the existence of an ecological niche of influenza viruses within the region and hence highlighting the need for joint epidemiologic and virologic surveillance to monitor the evolution of influenza viruses.

## Introduction

Influenza viruses are considered a major public health problem worldwide due to their potential to cause pandemics and yearly epidemics with considerable morbidity and significant mortality, with more than 250,000 deaths per year occurring worldwide due to influenza epidemics [1]. The genome of these viruses consists of eight segments of negative-sense single-stranded Ribonucleic Acid (RNA) [2,3]. The virus surface glycoproteins, hemagglutinin (HA) and neuraminidase (NA), have the highest evolutionary rates of all influenza proteins [2–4]. The amino acid substitutions which are accumulated in mutant viruses, enable the virus to evade the immune system [5–7]. This process of accumulation of amino acid substitutions can result in progressive antigenic changes in the surface glycoproteins known as antigenic drift [2]. In addition to point mutations, genetic reassortment also plays an important role in the evolution of newly emerging viruses [8,9].

The effectiveness of annually administered influenza vaccines relies on the selection of appropriate viruses that elicit optimal immunity against a wide range of influenza viruses circulating worldwide at that time [10,11]. Along with classical antigenic characterization, based on serological assays, sequencing of specific virus genes has become an integral surveillance tool that contributes to vaccine selection [12]. Permanent monitoring of the antigenic and genetic properties of locally circulating influenza viruses will help in directing local/regional vaccine needs and allow the monitoring of the (re)emergence of variant virus strains.

Limited data are available in sub-Saharan Africa and in regard to Mozambique, no data exist on antigenic, genetic profile of influenza virus. In this context, we conducted this study with the aim to assess the antigenic and genetic characteristics as well as susceptibility profile to antivirals of influenza viruses circulating in Mozambique. This study is part of the recently established Acute Respiratory Illnesses surveillance system in Mozambique [13,14].

## Materials and methods

### Ethics statement

This study was approved by the Mozambican National Bioethics Committee (IRB00002657). Verbal consent to participate was obtained from the legal representative of each child. This manuscript does not present any individual patient data, since all anonymized and coded, according to the influenza sentinel surveillance protocol.

### Study design and clinical specimens

As part of the WHO’s Global Influenza Surveillance and Response System (GISRS), the National Institute of Health (NIH) in Mozambique, established the influenza sentinel surveillance at two hospitals located in Maputo City, namely Mavalane General Hospital and Maputo Central Hospital.

Mozambique is situated in the southeast coast of Africa and has a tropical climate with two distinct seasons, the rainy season from November through April and the dry season for the rest of the year. Maputo city is the capital of Mozambique, with a total of 1.290.991 inhabitants.

During the 2015 season, the NIH received 1,140 nasopharyngeal and/or oropharyngeal swab specimens taken from inpatients with severe acute respiratory illness admitted at the two sentinel hospitals. The swabs were placed in 3 mL of virus transport medium and sent to the Virus Isolation Laboratory at the NIH on the same day of collection and were tested for influenza by OneStep real-time Reverse Transcriptase–Polymerase Chain Reaction (rtRT–PCR) using the Centers for Disease Control and Prevention (CDC, USA) influenza (typing and subtyping) protocol (S1 File). The positivity rate was 4.0% (46/1140).

For this study, 20 influenza (despite the type/subtype) positive specimens with cycle thresholds (Ct) below 30 were selected for antigenic and genetic analysis at the Worldwide Influenza Centre, Francis Crick Institute, United Kingdom, a World Health Organization (WHO) Collaborating Centre on Influenza (London WHO CC).

Of the 20 shipped specimens, 13 were positive for influenza A(H3N2), four for A(H1N1)pdm09 and three for type B viruses.

### Isolation of influenza virus

Viruses were recovered and propagated following inoculation of A(H1N1)pdm09 and type B-positive clinical specimens in conventional Madin–Darby canine kidney (MDCK) cells. A(H3N2)-positive specimens were inoculated in MDCK-SIAT1 cells (engineered to express increased levels of α-2,6-linked sialic acid receptors) [15]. Cells were maintained in Minimum Essential Medium with Earle’s salts, non-essential amino acids and L-glutamine (Gibco no. 41-500-034) and Dulbecco’s Modified Eagle’s Medium (Gibco no. 12 800–01) respectively, both supplemented with 2% fetal bovine serum (Gibco, USA). The presence of virus in the cell-culture supernatant was confirmed by hemagglutination activity (HA) according to WHO standard methods using suspensions of either guinea pig erythrocytes (1% v/v) or turkey erythrocytes (0.75% v/v) [16] or a sialidase assay using MUNANA [17].

### Antigenic characterization of influenza virus

Antigenic characterization of influenza isolates was performed by hemagglutination inhibition (HI) assays, following WHO standard methods [16]. For influenza A(H1N1pdm09) and influenza B viruses, 0.75% (v/v) suspensions of turkey red blood cells (RBCs) was used. For influenza A(H3N2) viruses 1% (v/v) guinea pig RBCs in the presence of oseltamivir carboxylate was used [18]. Panels of post-infection ferret antisera against vaccine and references virus strains were used to assess the antigenic characteristics of Mozambican strains. Viruses were considered antigenically similar or “like” each other if their HI titers differ by two dilutions or less [equivalent to a two-well (i.e. a four-fold dilution) or less difference] [16].

### Assessment of susceptibility of NA to oseltamivir and zanamivir

Susceptibility of viral NA to oseltamivir (Roche Diagnostics GmbH, Mannheim, Germany) and zanamivir (GlaxoSmithKline, Uxbridge, UK) was assessed by fluorescent neuraminidase activity inhibition [17]. The NA activity was measured using the fluorescent substrate, 2’-(4-methylumbelliferyl)-α-D-N-acetylneuraminic acid (MUNANA; Sigma, USA) and the inhibitor concentrations ranged from 0.03 nmol/L to 1,000 nmol/L. Briefly, a total volume of 45 μL containing 15 μL of the virus and 66 μM MUNANA in 21.66 mM MES buffer (pH 6.5) containing 2.66 mM CaCl_2_ was incubated for 60 minutes at 37 °C, and the reaction was stopped by addition of 150 μL of 0.14 M NaOH in 83% ethanol. The fluorescence of the released 4-methylumbelliferone sodium salt was measured at excitation and emission wavelengths of 355/365 nm and 450/460 nm, respectively. The activity of each virus sample was titrated, by assaying serial two-fold dilutions. Virus suspensions were adjusted to equivalent NA activities, which fell into the linear portion of the activity curve and the viruses were pre-incubated for 30 min at 37 °C with oseltamivir or zanamivir at final concentrations of 5 μM to 0.05 pM, in serial ten-fold dilutions.

### Genetic characterization and phylogenetic analysis of influenza virus HA and NA

For RT-PCR, virus RNA was extracted from 140 μL of infected cell culture supernatant or clinical sample using a QIAamp Viral RNA mini kit (QIAGEN), as described by the manufacturers. The extracted RNA was retro transcribed to complementary DNA (cDNA) using Invitrogen SuperScript III reverse transcriptase (Invitrogen) and subsequently amplified with Platinum Pfx DNA Polymerase (Thermo Life Sciences). Sanger dideoxy-sequencing was performed using a Big Dye Terminator Cycle sequencing kit (Applied Biosystems) and analysed on an ABI-3730XL DNA analyzer. Sequences were assembled and edited using the Staden software package and alignments were conducted in BioEdit. Phylogenetic analyses and comparisons with reference sequences were performed using RAxML, with the Annotator program used to indicate nodes defined by specific amino acid substitutions, and trees were drawn using FigTree version 1.4.3. The description and history of the biological effect of the identified amino acid substitutions (classical numbering) were adduced through the Global Initiative on Sharing All Influenza Data (GISAID)-associated link to FluSurver [19]. All sequences generated in the course of the present study were deposited in GISAID under the following identifiers EPI_ISL_(197469, 197478, 200227, 200228, 201663, 201667, 201668 201669, 201670, 201671, 201673, 201675, 201676, 201677, 201678, 201683, 201732 201741, 201744).

## Results

### General characteristics of participants

The 20 specimens that we selected in this study based on Ct value (Ct <30), represented 43.5% (20/46) of the total of influenza positive clinical specimens taken from hospitalized children with a severe acute respiratory illness who were enrolled into the influenza sentinel surveillance system at two hospitals during 2015 season. The general characteristics of the participants from whom these samples were taken are described in Table 1. The median age of the 20 children was 2 (0–12) years old, and 50% (10/20) were female. Among the 17 children with clinical diagnosis at the admission, bronchopneumonia and shortness of breath were the most frequent, with 65% (11/17) and 24% (4/17), respectively. Among these children, cough was the most common symptom with 95% (19/20), followed by difficulty breathing and fever with frequencies of 65% (13/20) and 40% (8/20), respectively. Asthma was observed in 20% of the patients and was the only underlying medical condition. Of the 11 children whose outcome was recorded, only one deceased.

**Table 1:** General characteristics of the participants analyzed in this study.

Characteristic		Frequency	
		n	%
**Gender (N = 20) **	Male	10	50.0
	Female	10	50.0
**Age, median (min- max)**	2 (0–12) years		
**Admission diagnosis (N = 17)**	Bronchopneumonia	11	64.7
	Shortness of breath	4	23.5
	Cough	1	5.9
	Febrile convulsions	1	5.9
	Bronchitis/Bronchiolitis	0	0.0
**Symptoms (N = 20)**	Cough	19	95.0
	Difficult breathing	13	65.0
	Coryza	12	60.0
	Fever (measured and history)	8	40.0
	Sore throat	2	10.0
	Headache	1	5.0
	Malaise	1	5.0
**Outcome (N = 11) **	Discharge	10	90.9
	Death	1	9.1

N- the number of patients with a record in each characteristic.

### Virus isolation and identification of the influenza virus type and subtype

Of the 20 specimens sent to the Francis Crick Institute, influenza virus was successfully recovered from 19 specimens, yielding a recovery rate of 95.0% (19/20). The only sample that was not recovered belonged to A(H3N2) subtype. All influenza B (n = 3) and A(H1N1)pdm09 (n = 4) viruses showed hemagglutination activity, but only five out of 12 (41.7%) recovered A(H3N2) viruses showed hemagglutination activity. However, all A(H3N2) viruses (n = 12) were confirmed by sialidase assay.

### Antigenic and genetic characterization of the viruses

#### Influenza A(H1N1)pdm09 viruses

All tested A(H1N1)pdm09 viruses (n = 4) were efficiently recognised by all post-infection ferret antisera which included antisera raised against the reference virus belonging to the genetic group 6B (A/South Africa/3626/2013) and the vaccine virus (A/California/7/2009) recommended for the Southern and Northern Hemispheres for 2016 and 2016/2017. The exception was that the antiserum raised against A/Christchurch/16/2010 recognised only one Mozambican isolate at a titre within 4-fold of the homologous titre (Table 2).

**Table 2:** Antigenic (HI) analyses of A(H1N1)pdm09 viruses.

				Hemagglutination inhibition titre								
				Post-infection ferret antisera								
Viruses	Genetic group	Collection date	Reference viruses	A/Cal 7/09	A/Bayern 69/09	A/Lviv N6/09	A/Chch 16/10	A/Astrak 1/11	A/St. P 27/11	A/St. P 100/11	A/HK 5659/12	A/Sth Afr 3626/13
			Ferret number	F05/14	F09/15	F14/13	F15/14	F22/13	F26/14	F24/11	F30/12	F3/14
			Genetic group				4	5	6	7	6A	6B
Reference viruses			Passage History*									
A/California/7/2009		2009-04-09	E1/E3	***640***	640	640	160	160	160	320	160	160
A/Bayern/69/2009		2009-07-01	MDCK5/MDCK1	160	***640***	320	80	80	40	40	80	80
A/Lviv/N6/2009		2009-10-27	MDCK4/SIAT1/MDCK3	320	640	***640***	160	80	80	80	160	80
A/Christchurch/16/2010	4	2010-07-12	E1/E3	640	640	1280	***2560***	1280	640	1280	2560	640
A/Astrakhan/1/2011	5	2011-02-28	MDCK1/MDCK5	640	320	320	320	***1280***	640	1280	2560	640
A/St. Petersburg/27/2011	6	2011-02-14	E1/E3	2560	1280	1280	640	2560	***1280***	5120	2560	1280
A/St. Petersburg/100/2011	7	2011-03-14	E1/E3	1280	640	640	640	1280	640	***2560***	2560	640
A/Hong Kong/5659/2012	6A	2012-05-21	MDCK4/MDCK2	160	160	80	80	320	160	640	***640***	320
A/South Africa/3626/2013	6B	2013-06-06	E1/E3	320	320	320	320	640	320	640	640	***640***
**Test viruses**												
A/Mozambique/IR418/2015	6B	2015-01-21	MDCK1	640	640	320	320	640	320	1280	1280	640
A/Mozambique/IR467/2015	6B	2015-02-13	SIAT1	320	320	320	320	640	320	1280	640	320
A/Mozambique/IR495/2015	6B	2015-02-19	MDCK1	320	640	320	320	640	320	640	640	640
A/Mozambique/IR543/2015	6B	2015-03-03	MDCK1	640	640	320	640	1280	640	2560	1280	1280

*E–Egg; MDCK–Madin–Darby Canine Kidney; SIAT–(MDCK-SIAT1 cells engineered to express increased levels of α-2,6-linked sialic acid receptors); the number of passages required to generate isolate/produce sufficient virus for HA/HI analyses is indicated behind each host/cell line used; Homologous titres are indicated by underlined bold italics.

**Fig 1:**
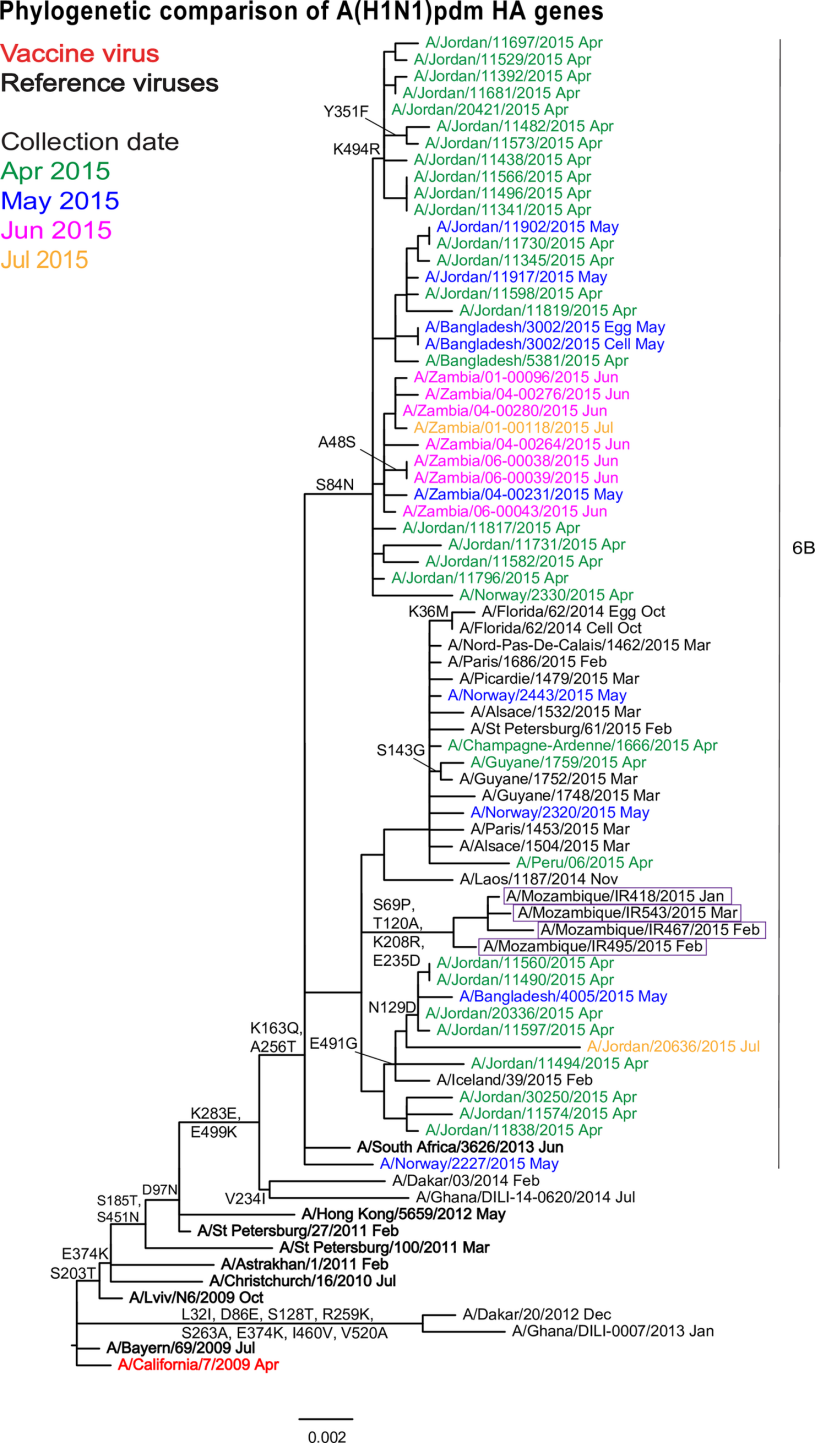
Phylogenetic comparison of influenza A(H1N1)pdm09 HA genes. The month of clinical specimen collection is indicated by colour (April to July 2015) and after each virus name. Specific viruses are highlighted: vaccine virus (bold red), reference viruses to which post-infection ferret antisera were raised (bold black) and Mozambican viruses (boxed). Amino acid substitutions defining specific genetic clusters are indicated at nodes and virus-specific substitutions are shown after the virus name (* indicates polymorphism). Genetic group 6B, defined by HA1 amino acid substitutions K163Q and A256T, is indicated and the scale bar indicates the distance between isolates.

Hemagglutinin phylogenetic analysis also showed that A(H1N1)pdm09 viruses were similar to viruses circulating globally and belonging to genetic sub-group 6B defined by amino acid substitutions D97N, S185T, S203T and K283E in HA1 and E374K, S451N and E499K in HA2 (characteristic of group 6) and additional HA1 amino acid substitutions K163Q and A256T (characteristic of sub-group 6B) (Fig 1). The Mozambican isolates clearly clustered together in the phylogenetic tree and were defined by an unusual set of HA1 amino acid substitutions: S69P, T120A, K208R and E235D (S2 Table).

A similar topology was observed when analysing the NA sequences which contained R257K and I288V amino acid substitutions, defining genetic sub-group 6B (S1 Fig).

### Influenza A(H3N2) viruses

All A(H3N2) viruses (n = 12) were successfully propagated in MDCK-SIAT1 cells, but only five were able to agglutinate guinea pig RBCs in the presence of oseltamivir. As shown in Table 3, all five Mozambican viruses were well recognised by antiserum raised against cell culture-propagated A/Hong Kong/5738/2014 (subclade 3C.2a virus), at titres within 2-fold of the homologous titre. But antiserum raised against the other subclade 3C.2a virus, egg-propagated A/Hong Kong/4801/2014 recognised the five Mozambican viruses at different titres: two at titres within 2-fold, other two at 4-fold and one virus at 8-fold reductions compared to the titres of the antisera for the homologous virus. Antiserum raised against egg-propagated A/Stockholm/6/2014, a virus from subclade 3C.3a showed similar reactivity profile as antiserum raised against A/Hong Kong/4801/2014. Antiserum raised against cell culture-propagated A/Stockholm/6/2014 recognised all five test viruses at titres 2-fold reduced compared to the titre with the homologous virus. Antiserum raised against another 3C.3a subclade virus, egg-propagated A/Switzerland/9715293/2013, previously recommended for use in vaccines, showed low dynamic range, recognising test viruses at homologous titres ≤ 80. All test viruses were poorly recognised by antisera raised against viruses from clades previously in circulation (A/Texas/50/2012, 3C.1; A/Hong Kong/146/2013, 3C.2 and A/Samara/73/2013, 3C.3) and A/Netherland/525/2014, 3C.3b included.

**Table 3:** Antigenic (HI) analyses of A(H3N2) viruses (guinea pig RBCs in the presence of 20 nM oseltamivir).

				Haemagglutination inhibition titre									
				Post-infection ferret antisera									
Viruses	Genetic group	Collection Date	Reference viruses	A/Texas 50/12	A/Samara 73/13	A/HK 146/13	A/Stock 6/14	A/Stock 6/14	A/Switz 9715293/13	A/Switz 9715293/13	A/HK 5738/14	A/Neth 525/14	A/HK 4801/14
			Ferret number	F36/12	F24/13	F10/15	F14/14	F20/14	F13/14	F32/14	F30/14	F23/15	F12/15
			Genetic group	3C.1	3C.3	3C.2	3C.3a	3C.3a	3C.3a	3C.3a	3C.2a	3C.3b	3C.2a
Reference viruses			Passage History*										
A/Texas/50/2012	3C.1	2012-04-15	E5/E2	***5120***	640	320	160	640	40	640	160	320	80
A/Hong Kong/146/2013	3C.2	2013-01-11	E3/E3	2560	640	***640***	80	640	40	640	320	320	160
A/Hong Kong/4801/2014	3C.2	2014-02-26	E6/E1 isolate 1	80	160	40	160	160	40	40	320	80	***320***
A/Netherlands/525/2014	3C.2	2014-12-17	SIAT2/SIAT3	640	320	160	320	160	80	160	80	***1280***	160
A/Samara/73/2013	3C.3	2013-03-12	C1/SIAT3	1280	***640***	320	320	320	80	320	320	320	160
A/Hong Kong/5738/2014	3C.2a	2014-04-30	MDCK1/MDCK3	80	160	80	320	160	40	80	***160***	40	160
A/Switzerland/9715293/2013	3C.3a	2013-12-06	SIAT1/SIAT2	40	80	<	320	160	***80***	80	80	<	40
A/Switzerland/9715293/2013	3C.3a	2013-12-06	E4/E1 clone 123	320	160	80	320	320	80	***640***	160	40	80
A/Stockholm/6/2014	3C.3a	2014-02-06	E4/E1 isolate 2	640	80	80	160	***320***	80	640	160	40	40
A/Stockholm/6/2014	3C.3a	2014-02-06	SIAT1/SIAT2	160	320	80	***320***	160	160	160	80	40	80
**Test viruses**													
A/Mozambique/IR424/2015	3C.2a	2015-01-26	SIAT1	160	160	80	160	160	40	80	160	40	160
A/Mozambique/IR436/2015	3C.2a	2015-01-30	SIAT1	80	80	40	160	160	<	40	160	<	160
A/Mozambique/IR451/2015	3C.2a	2015-02-09	SIAT1	80	80	40	160	80	<	40	160	<	80
A/Mozambique/IR493/2015	3C.2a	2015-02-19	SIAT1	80	80	40	160	80	<	40	80	<	80
A/Mozambique/IR803/2015	3C.2a	2015-05-06	SIAT2	40	80	40	160	40	<	40	80	<	40

*E–Egg; C–identity of cell line unknown; MDCK–Madin–Darby Canine Kidney; SIAT–(MDCK-SIAT1 cells engineered to express increased levels of α-2,6-linked sialic acid receptors); the number of passages required to generate isolate/produce sufficient virus for HA/HI analyses is indicated behind each host/cell line used. Homologous titres are indicated by underlined bold italics; < refers titre of below 40.

**Fig 2:**
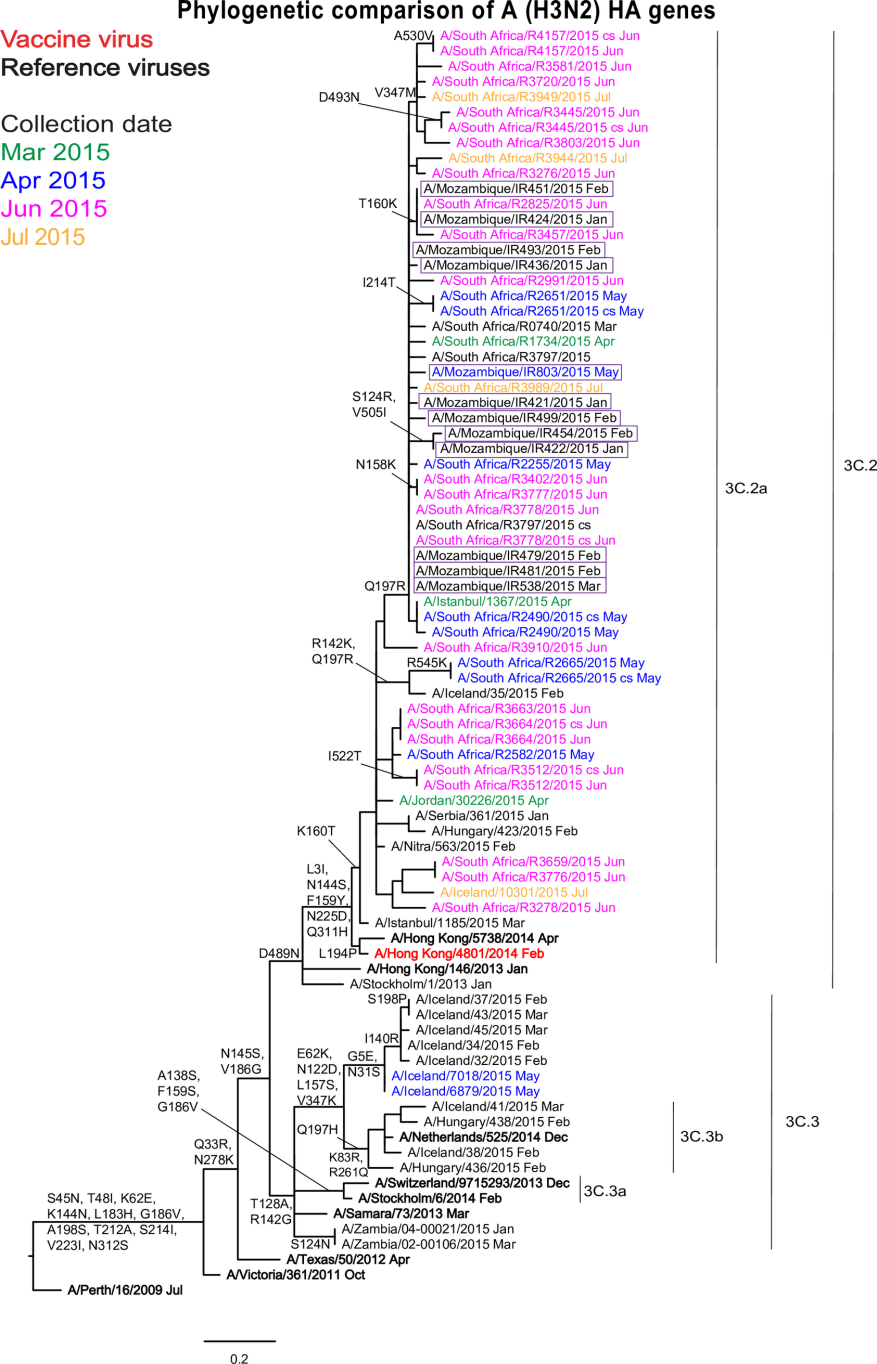
Phylogenetic comparison of influenza A(H3N2) HA genes. The month of clinical specimen collection is indicated by colour (March to July 2015) after each virus name. Specific viruses are highlighted: vaccine virus (bold red), reference viruses to which post-infection ferret antisera were raised (bold black) and Mozambican viruses (boxed). Amino acid substitutions defining specific genetic clusters are indicated at nodes and virus-specific substitutions are shown after the virus name (* indicates polymorphism). Genetic clades and subclades are indicated at the right of the tree and the scale bar indicates the distance between isolates.

Hemagglutinin phylogenetic analysis showed that all 12 Mozambican influenza A(H3N2) strains, including those unable to be analysed by HI, clustered with viruses circulating globally in subclade 3C.2a (Fig 2). This subclade includes the currently recommended vaccine virus A/Hong Kong/4801/2014. The subclade is defined by amino acid substitutions N145S in HA1 and D489N in HA2 (clade 3C.2) and additional HA1 substitutions resulting in the loss (L3I, N144S) and gain (F159Y, K160T, N225D) potential N-linked glycosylation sites and Q311H. All Mozambican strains isolated between January and May grouped with South African strains isolated between May and July. This group was defined by amino acid substitution in HA1 Q197R.

A similar topology was observed when analysing the NA sequences (S2 Fig). Two Mozambican isolates showed HA1 T160K reversion and two other isolates carried HA1 S124R and HA2 V505I substitutions (Fig 2, S3 Table). Interestingly, of the five strains that could be analysed antigenically by HI, four showed loss or partial loss of the 158–160 glycosylation motif due to HA1 T160K or T160I substitution or T160 polymorphism (T160).

### Influenza B viruses

All three influenza B virus isolates from Mozambique were successfully propagated and showed HI activity and were recognised well by the hyper-immune sheep antiserum raised against the vaccine virus egg-propagated B/Phuket/3073/2013. Influenza B/Yamagata lineage viruses with HA genes from clade 2 (the B/Massachusetts/02/2012 clade) can be differentiated by some antisera from those with HA genes in clade 3 (the B/Wisconsin/1/2010 –B/Phuket/3073/2013 clade). As shown in Table 4, the antiserum raised against egg-propagated B/Hong Kong/3417/2014 (a clade 3 virus) recognised all three test viruses at titres equal to its homologous titre but this antiserum recognises reference viruses from clade 2 and clade 3 at titres within 2-fold of each other. Two of the three test viruses, B/Mozambique/IR981/2015 and B/Mozambique/IR1062/2015 were recognised by antiserum raised against egg-propagated B/Phuket/3073/2013, at titres within 2-fold of the homologous virus titre, and likewise the antiserum raised against the previous vaccine virus egg-propagated B/Wisconsin/1/2010 recognised B/Mozambique/IR981/2015 and B/Mozambique/IR1062/2015 at titres within 2-fold of the homologous titre of the antiserum, B/Mozambique/IR1010/2015 being recognised somewhat less well by both these antisera at titres 4-fold lower than the homologous titres of these antisera. B/Mozambique/IR981/2015 and B/Mozambique/IR1062/2015 were recognised by antiserum raised against the cell culture-propagated B/Phuket/3073/21013, but at titres within 4-fold of its homologous titre, and B/Mozambique/IR1010/2015 was recognised poorly by this antiserum. Surprisingly, the antiserum raised against egg-propagated B/Stockholm/1/2011 recognised all three test viruses poorly. An antiserum raised against egg-propagated clade 2 vaccine virus B/Massachusetts/02/2012 recognised B/Mozambique/IR981/2015 and B/Mozambique/IR1062/2015 at titres within 4-fold of its homologous titre but the antiserum raised against the cell culture-propagated cultivar of B/Massachusetts/02/2012 recognised only B/Mozambique/IR981/2015 at a titre within 4-fold of its homologous titre. The antiserum raised against cell culture-propagated B/Estonia/55669/2011 (a clade 3 virus) had a low homologous titre of 80 and recognised B/Mozambique/IR981/2015 and B/Mozambique/IR1062/2015 at titres within 2-fold and 4-fold, respectively, of the titre of the antiserum for the homologous virus and recognised B/Mozambique/IR1010/2015 poorly. Overall antisera raised against viruses from clade 3 recognised the test viruses from Mozambique somewhat better than the antisera raised against viruses from clade 2.

**Table 4:** Antigenic (HI) analyses of influenza B viruses.

				Haemagglutination inhibition titre								
				Post-infection ferret antisera								
Viruses	Genetic group	Collection date	Reference viruses	B/Phuket 3073/13	B/Esto 55669/11	B/Mass 02/12	B/Mass 02/12	B/Wis 1/10	B/Stock 12/11	B/Phuket 3073/13 egg	B/Phuket 3073/13 cell	B/HK 3417/13
			Ferret number	SH614^$^	F32/12	F42/14	F15/13	F10/13	F06/15	F36/14	F35/14	F715/14
			Genetic group	3	2	2	2	3	3	3	3	3
Reference viruses			Passage History*									
B/Florida/4/2006	1	2006-12-15	E7/E1	1280	40	640	80	160	160	160	40	160
B/Brisbane/3/2007	2	2007-09-03	E2/E3	1280	40	640	80	160	160	160	20	160
B/Estonia/55669/2011	2	2011-03-14	MDCK2/ MDCK3	1280	***80***	80	320	40	<	80	40	160
B/Massachusetts/02/2012	2	2012-03-13	E3/E3	1280	40	***320***	160	160	80	160	20	160
B/Massachusetts/02/2012	2	2012-03-13	MDCK1/C2/ MDCK3	1280	160	640	***320***	160	80	160	80	320
B/Wisconsin/1/2010	3	2010-02-20	E3/E3	2560	20	160	40	***160***	80	160	40	320
B/Stockholm/12/2011	3	2011-03-28	E4/E1	1280	<	160	40	80	***160***	80	40	160
B/Phuket/3073/2013	3	2013-11-21	E4/E2	***2560***	20	160	40	160	80	***160***	40	320
B/Phuket/3073/2013	3	2013-11-21	MDCK2/ MDCK2	***5120***	160	160	160	160	80	320	***320***	320
B/Hong Kong/3417/2014	3	2014-06-04	E4/E1	1280	<	80	40	80	<	80	20	***160***
**Test viruses**												
B/Mozambique/IR981/2015	3	2015-06-08	MDCK2	2560	40	80	80	80	<	160	80	160
B/Mozambique/IR1010/2015	3	2015-06-11	MDCK1	1280	<	40	20	40	20	40	20	160
B/Mozambique/IR1062/2015	3	2015-06-23	MDCK2	2560	20	80	40	80	<	80	80	160

* E–Egg; C–the identity of cell line unknown; MDCK–Madin–Darby Canine Kidney; the number of passages required to generate isolate/produce sufficient virus for HA/HI analyses is indicated behind each host/cell line used. Homologous titres are indicated by underlined bold italics; < refers to titre below 20. ^$^—hyper-immune sheep antiserum.

**Fig 3:**
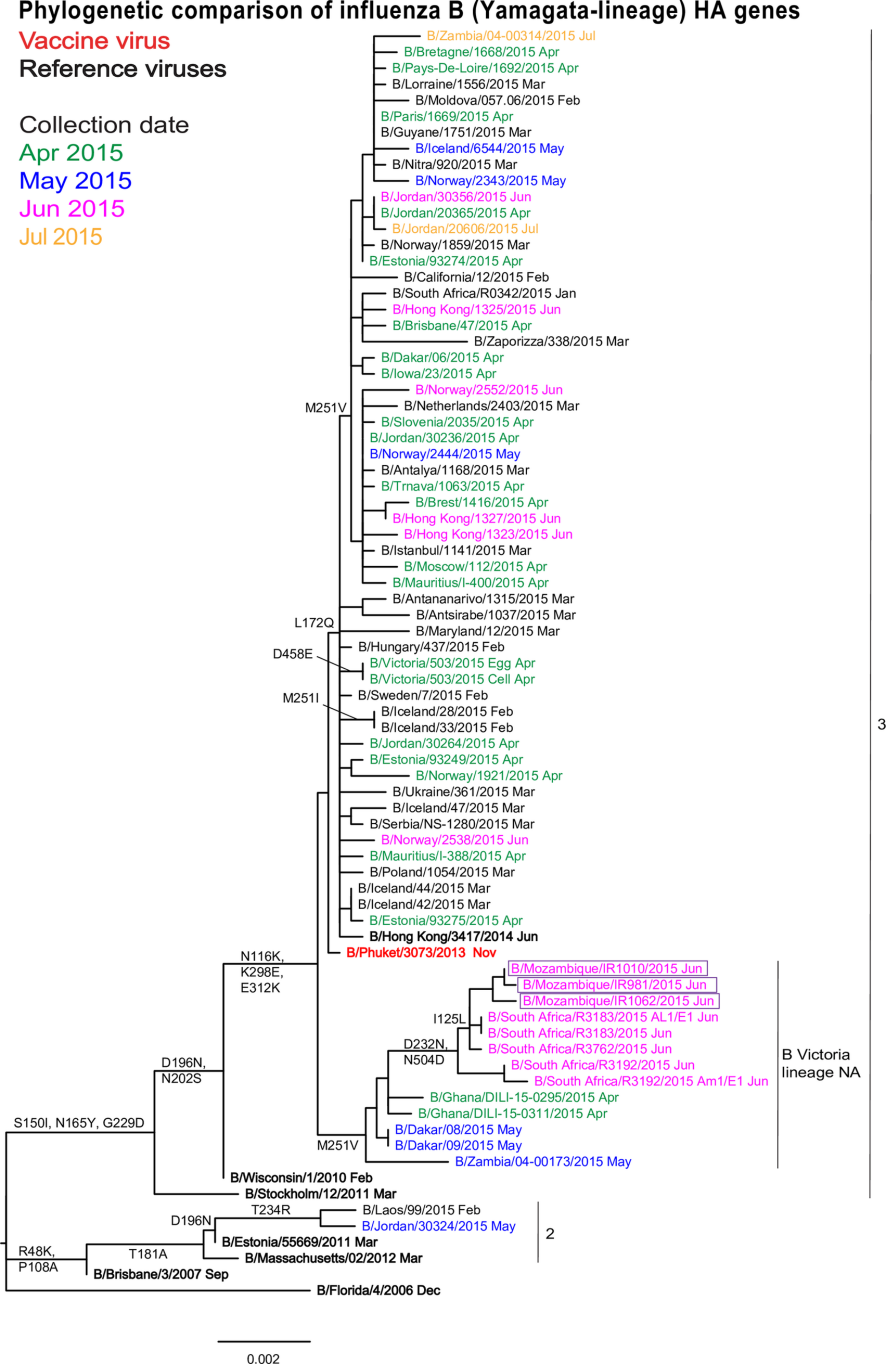
Phylogenetic comparison of influenza B/Yamagata-lineage HA genes. The month of clinical specimen collection is indicated by colour (April to July 2015) and after each virus name. Specific viruses are highlighted: vaccine virus (bold red), reference viruses to which post-infection ferret antisera were raised (bold black) and Mozambican viruses (boxed). Amino acid substitutions defining specific genetic clusters are indicated at nodes and virus-specific substitutions are shown after the virus name (*indicates polymorphism). Genetic clades are indicated to the right of the tree and the scale bar indicates the distance between isolates.

All three Mozambican influenza B strains had HA genes that fell into genetic clade 3 of the B/Yamagata-lineage along with the 2016 vaccine virus B/Phuket/3073/2013 (Fig 3). However, these isolates carried NA genes of the B/Victoria-lineage also observed in other viruses from Africa (Ghana, Senegal, South Africa and Zambia) with which they formed a group in the phylogenetic tree characterized by HA1 M251V amino acid substitution. Mozambique isolates formed a distinct subgroup with South African isolates carrying additional amino acid substitutions in HA1 (I125L and D232N) and HA2 (N504D) (S4 Table).

### Antiviral susceptibility

Phenotypic analysis to assess virus susceptibility to oseltamivir and zanamivir (antivirals) was performed on all 19 test viruses that showed NA sialidase activity. All test viruses showed normal inhibition by both NA inhibitors (S1 Table).

## Discussion

Monitoring of the genetic and antigenic profile of influenza viruses is crucial for tracking the mutations that may affect the virulence, antigenicity, antiviral susceptibility and other phenotypic traits in order to guide an effective national and global influenza prevention and control strategies. This also serves as a global alert mechanism for the emergence of influenza viruses with pandemic potential [16,20].

In this study, we report results of virologic and drug susceptibility surveillance of representative influenza A(H1N1)pdm09, A(H3N2) and B viruses in Mozambique for the first time. This data contributes to the limited evidence available on this matter in sub-Saharan Africa.

The four Mozambican influenza A(H1N1)pdm09 strains tested were antigenically very similar to A/California/7/2009 and fell into genetic group 6B that had been in widespread circulation between 2013 and 2016 when sub-group 6B.1 viruses became predominant around the world [21–23]. When compared to the reference sequence (A/California/7/2009 virus), a number of nucleotides substitutions was detected in the Mozambican A(H1N1)pdm09 strains HA sequences. As adduced from Fluserver [19], of the substitutions detected in the Mozambican HA sequences, P83S, D97N, K163Q, S185T, S203T are known to be the most frequent and globally distributed (detected in more than 18% of the global HA sequences in more than 24 countries) while the substitutions S69P, T120A, K208R and E235D are known to be the less frequently distributed globally (detected in less than 1% of global HA sequences). Of note, all the substitutions observed in the Mozambican HA sequences were also observed in the 2015 South African HA sequences used in this study. No biological effect of the detected substitutions was elucidated or described in the literature. However, the substitutions P83S, S185T and S203T have been observed since the 2009/2010 and were considered variant markers of the influenza A(H1N1)pdm09 viruses during the 2009 pandemic [24,25]. Of note, none of the HA substitutions known to affect antigenic characteristics and/or disease severity (D222E, S185N, Q293H in HA1, N154I and I277V in HA2) [26] were observed in this study. In addition, more than 10 amino acid substitutions in NA sequences were observed in Mozambican isolates (S1 Fig). However, the sensitivity of these isolates to oseltamivir and zanamivir was not affected.

As previously reported, more than half of the Mozambican influenza A(H3N2) isolates failed to agglutinate all RBCs. This loss of the ability to agglutinate RBCs and poor replicative capacity in MDCK cells have been reported worldwide as a result of low binding affinities of the viral HA molecules to their receptors [22,23,27]. Substitutions at positions 159 and 225 observed in Mozambican HA sequences may have affected receptor-binding of viruses because these mutations have been associated with a decreased receptor-binding [27,28]. To overcome the difficulties in the antigenic characterization of A(H3N2) viruses by HI assay, NA-mediated agglutination, HA and HI assays were performed using guinea pig RBCs in the presence of 20 nM oseltamivir [29].

All five A(H3N2) Mozambican viruses, that were analyzed by HI assays, fell in genetic subclade 3C.2a and showed antigenic and genetic similarity to reference viruses from Hong Kong isolated in 2014, including the A/Hong Kong/4801/2014 strain, recommended for use in vaccines for the Southern Hemisphere 2016 and 2017 and Northern Hemisphere 2016/2017 and 2017/2018 influenza seasons [21,30,31]. Antiserum raised against cell culture-propagated A/Hong Kong/5738/2014 showed better results than antiserum raised against egg-cultured A/Hong Kong/4801/2014, which recognized less than half of Mozambican A(H3N2) isolates and only 53% of viruses from a range of countries [22]. The observed reduced recognition of Mozambican and world isolates by antiserum raised against vaccine strain may be related to egg-adaptive changes known to affect virus antigenicity [32–35].

The genetic clade 3 virus, B/Hong Kong/3417/2014, would be the best choice as a vaccine component for Mozambique, as the antiserum raised against it recognised well all the three Mozambican isolates than the antisera raised against another genetic clade 3 strain B/Phuket/3073/2013 that was recommended vaccine component for 2016 and 2017 Southern Hemisphere influenza season and 2016/2017 Northern Hemisphere influenza season [21,31] that poorly recognised one of the three Mozambican viruses. However, antigenic analysis of global influenza B viruses isolates showed that antiserum raised against the egg-propagated vaccine virus B/Phuket/3073/2013, recognised 100% of test viruses collected after 2015-08-31 at titres within 4-fold of the titre with the homologous virus [22].

Genetic analysis supported the antigenic analysis since all Mozambican isolates fell into the B/Phuket/3073/2013 clade. Interestingly, Mozambican HA sequences formed a sub-cluster with other African isolates carrying the HA1 M251V amino acid substitution. This sub-cluster also contained NA genes from the influenza B/Victoria-lineage. According to WHO CC London reports, reassortant viruses were sporadically seen in various parts of the world [22,23], but no literature was found describing the origin of those reassortant viruses. However, the continuous co-circulation of influenza B lineages was not observed during 2013 and 2014 seasons in South Africa[36]. Additionally, Mozambican isolates were more closely related to isolates from South Africa isolated in the same month as the Mozambican. That subcluster formed by Mozambique and South Africa carried additional substitutions (I125L and D232N in HA1 and N504D in HA2). These substitutions, with the exception of I125L, have been observed across many countries and have no known marked biological effect. However, as adduced from FluServer, they have been associated with HA oligomerization interfaces, host cell receptor-binding and antibody recognition sites [19]. An unusual substitution (T37A) in HA1 was observed in only one Mozambican isolate. Analysis performed from FluServer (GISAID database) revealed that this substitution has been rarely reported since December 2013 [19] and again, no marked biological effect had been described.

All isolates examined were more closely related to viruses circulating in the Southern African Region, regardless of transmission zones defined by WHO as geographical groups of countries, areas or territories with similar influenza transmission patterns [37]. This suggests an ecological niche of influenza virus within the region and thus, corroborate with the recently defined vaccination zones by WHO [38].

We would like to acknowledge few limitations of this study such as the small number of influenza virus isolates and the incomplete clinical and demographic data records; It has somehow limited the analysis of clinical outcomes with genetic characteristics.

## Conclusion

Overall, all Mozambican isolates, both influenza A and B subtypes showed high antigenic and genetic similarity to African isolates and those recommended for season vaccines. Additionally, findings from this study highlight the importance and the need of permanent: (i) sharing influenza-positive samples, (ii) performing regular virologic and epidemiological surveillance and (iii) performing antigenic and genetic evolution analysis of influenza viruses at country and regional levels. Outcomes from such actions will contribute to the design of effective control measures in the region, including the use of effective vaccines. Further studies should be conducted to evaluate the circulation and evolutionary patterns of the virus within the Southern Africa region.

## Supporting information

S1 Fig Phylogenetic comparison of influenza A(H1N1)pdm09 NA genes. The month of clinical specimen collection is indicated by colour (April to July 2015) and after each virus name. Specific viruses are highlighted: vaccine virus (bold red), reference viruses to which post-infection ferret antisera were raised (bold black) and Mozambican viruses (boxed). Amino acid substitutions defining specific genetic clusters are indicated at nodes and virus-specific substitutions are shown after the virus name (* indicates polymorphism). Genetic group 6B is indicated and the scale bar indicates the distance between isolates.
(TIF)

S2 Fig Phylogenetic comparison of influenza A(H3N2) NA genes. The month of clinical specimen collection is indicated by colour (March to July 2015) and after each virus name. Specific viruses are highlighted: vaccine virus (bold red), reference viruses to which post-infection ferret antisera were raised (bold black) and Mozambican viruses (boxed). Amino acid substitutions defining specific genetic clusters are indicated at nodes and virus-specific substitutions are shown after the virus name (* indicates polymorphism). Genetic clades and subclades are indicated to the right of the tree and the scale bar indicates the distance between isolates.
(TIF)

S1 Table Neuraminidase inhibitors susceptibility of Mozambican influenza virus. Susceptibility of viral NA to oseltamivir (Roche Diagnostics GmbH, Mannheim, Germany) and zanamivir (GlaxoSmithKline, Uxbridge, UK) was assessed by fluorescent neuraminidase activity inhibition. The NA activity was measured using the fluorescent substrate, 2’-(4-methylumbelliferyl)-α-D-N-acetylneuraminic acid (MUNANA; Sigma, USA) and the inhibitor concentrations ranged from 0.03 nmol/L to 1,000 nmol/L.
(DOC)

S2 Table Amino acids substitution in Mozambican influenza A(H1N1)pdm09 HA sequences and those with whom they clustered and reference sequences in the tree using A/California/7/2009 as a reference. (DOC)

S3 Table Amino acid substitutions in Mozambican influenza A(H3N2) HA sequences and those with whom they clustered and reference sequences in the tree using A/Perth/16/2009 as a reference. (DOC)

S4 Table Amino acids substitution in Mozambique influenza B HA sequences and those with whom they clustered and reference sequences in the tree using B/Florida/4/2006 as a reference. (DOC)

S1 File The CDC protocol for influenza virus typing and subtyping. (PDF)
