# Supplementary figures and images for: Antigenic and genetic characterization of influenza viruses isolated in Mozambique during the 2015 season
Source: PLoS One. 2018 Jul 26;13(7):e0201248. doi: 10.1371/journal.pone.0201248 (PMC6062064; doi:10.1371/journal.pone.0201248)

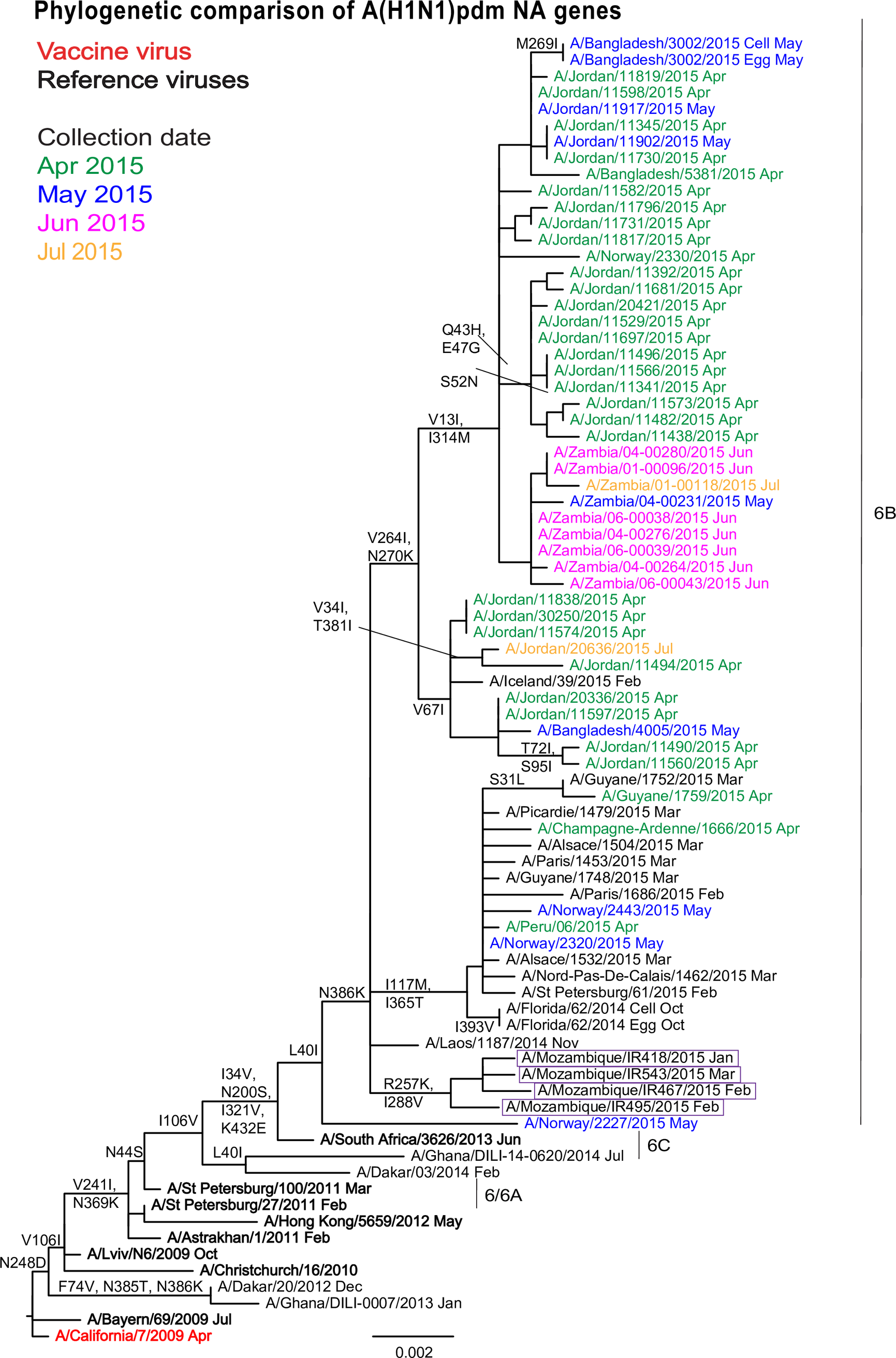

Supplement: S1 Fig — The month of clinical specimen collection is indicated by colour (April to July 2015) and after each virus name. Specific viruses are highlighted: vaccine virus (bold red), reference viruses to which post-infection ferret antisera were raised (bold black) and Mozambican viruses (boxed). Amino acid substitutions defining specific genetic clusters are indicated at nodes and virus-specific substitutions are shown after the virus name (* indicates polymorphism). Genetic group 6B is indicated and the scale bar indicates the distance between isolates. (TIF) [file pone.0201248.s001.tif]

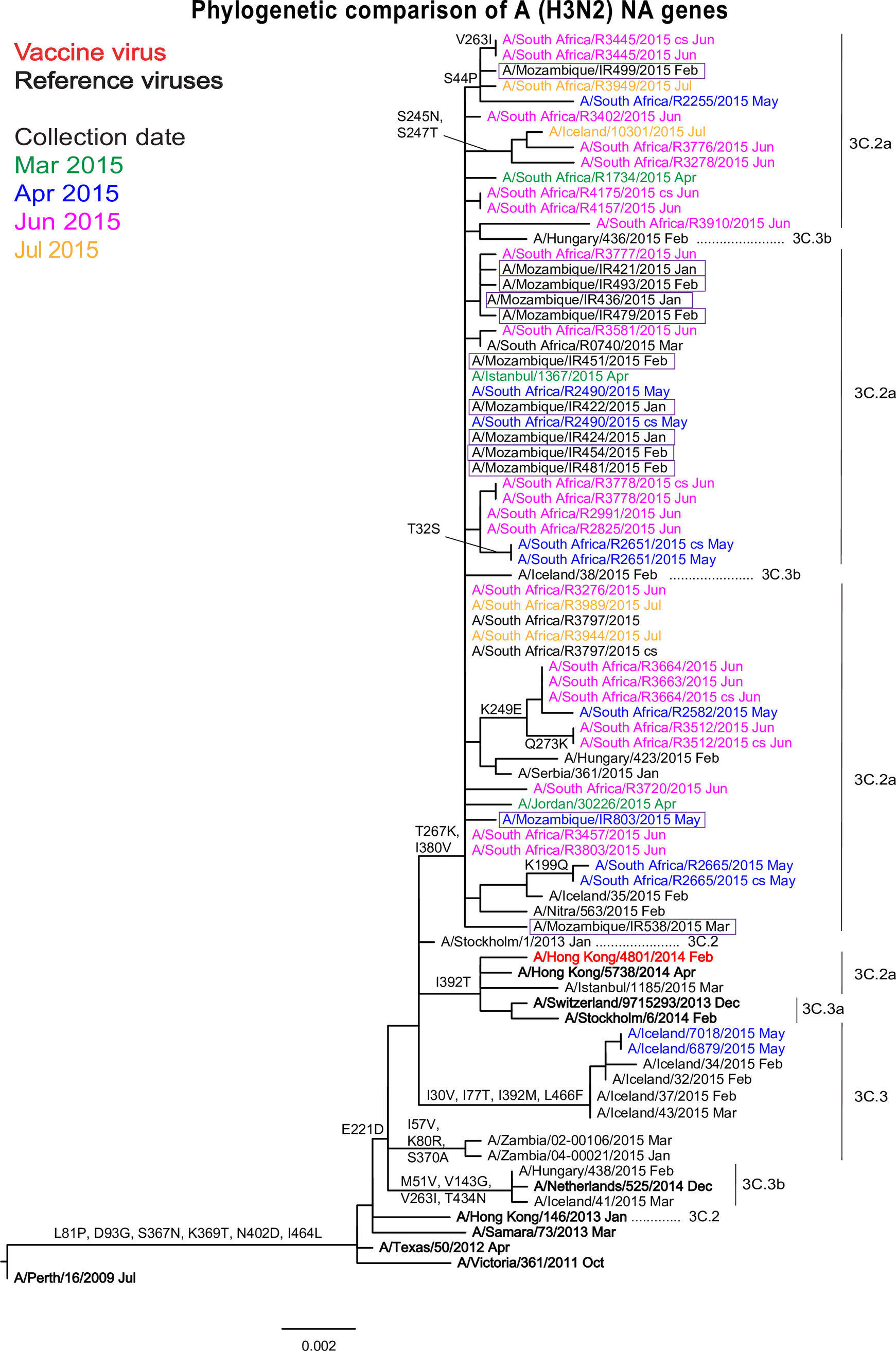

Supplement: S2 Fig — The month of clinical specimen collection is indicated by colour (March to July 2015) and after each virus name. Specific viruses are highlighted: vaccine virus (bold red), reference viruses to which post-infection ferret antisera were raised (bold black) and Mozambican viruses (boxed). Amino acid substitutions defining specific genetic clusters are indicated at nodes and virus-specific substitutions are shown after the virus name (* indicates polymorphism). Genetic clades and subclades are indicated to the right of the tree and the scale bar indicates the distance between isolates. (TIF) [file pone.0201248.s002.tif]
