# Supplementary material for: Antigenic and genetic characterization of influenza viruses isolated in Mozambique during the 2015 season
Source: PLoS One. 2018 Jul 26;13(7):e0201248. doi: 10.1371/journal.pone.0201248 (PMC6062064; doi:10.1371/journal.pone.0201248)
Supplement: S1 Table — Susceptibility of viral NA to oseltamivir (Roche Diagnostics GmbH, Mannheim, Germany) and zanamivir (GlaxoSmithKline, Uxbridge, UK) was assessed by fluorescent neuraminidase activity inhibition. The NA activity was measured using the fluorescent substrate, 2’-(4-methylumbelliferyl)-α-D-N-acetylneuraminic acid (MUNANA; Sigma, USA) and the inhibitor concentrations ranged from 0.03 nmol/L to 1,000 nmol/L. (DOC) [file pone.0201248.s003.doc]

**S1 Table 1. Neuraminidase inhibitors susceptibility of Mozambican influenza virus**

|  | | | | **Oseltamivir** | | | **Zanamivir** | |
| --- | --- | --- | --- | --- | --- | --- | --- | --- |
| **Virus name** | **Collection date (dd/mm/yyyy)** | **Type/Subtype** | **IC50** | | **Sensitivity** | **IC50** | | **Sensitivity** |
| A/Mozambique/IR418/2015 | 21/01/2015 | A(H1N1)pdm09 | 0.8 | | Normal inhibition | 0.42 | | Normal inhibition |
| A/Mozambique/IR467/2015 | 13/02/2015 | A(H1N1)pdm09 | 1.1 | | Normal inhibition | 0.38 | | Normal inhibition |
| A/Mozambique/IR495/2015 | 19/02/2015 | A(H1N1)pdm09 | 1.07 | | Normal inhibition | 0.58 | | Normal inhibition |
| A/Mozambique/IR543/2015 | 03/03/2015 | A(H1N1)pdm09 | 0.77 | | Normal inhibition | 0.52 | | Normal inhibition |
| A/Mozambique/IR421/2015 | 26/01/2015 | A(H3N2) | 1.08 | | Normal inhibition | 0.55 | | Normal inhibition |
| A/Mozambique/IR422/2015 | 26/01/2015 | A(H3N2) | 0.33 | | Normal inhibition | 0.27 | | Normal inhibition |
| A/Mozambique/IR424/2015 | 26/01/2015 | A(H3N2) | 0.34 | | Normal inhibition | 0.34 | | Normal inhibition |
| A/Mozambique/IR436/2015 | 30/01/2015 | A(H3N2) | 0.53 | | Normal inhibition | 0.36 | | Normal inhibition |
| A/Mozambique/IR451/2015 | 09/02/2015 | A(H3N2) | 0.56 | | Normal inhibition | 0.33 | | Normal inhibition |
| A/Mozambique/IR454/2015 | 09/02/2015 | A(H3N2) | 0.48 | | Normal inhibition | 0.36 | | Normal inhibition |
| A/Mozambique/IR479/2015 | 17/02/2015 | A(H3N2) | 0.33 | | Normal inhibition | 0.22 | | Normal inhibition |
| A/Mozambique/IR481/2015 | 17/02/2015 | A(H3N2) | 0.37 | | Normal inhibition | 0.34 | | Normal inhibition |
| A/Mozambique/IR493/2015 | 19/02/2015 | A(H3N2) | 0.26 | | Normal inhibition | 0.29 | | Normal inhibition |
| A/Mozambique/IR499/2015 | 20/02/2015 | A(H3N2) | 0.36 | | Normal inhibition | 0.26 | | Normal inhibition |
| A/Mozambique/IR551/2015 | 04/03/2015 | A(H3N2) | not recovered | | | | | |
| A/Mozambique/IR538/2015 | 02/03/2015 | A(H3N2) | 0.3 | | Normal inhibition | 0.29 | | Normal inhibition |
| A/Mozambique/IR803/2015 | 06/05/2015 | A(H3N2) | 0.52 | | Normal inhibition | 0.33 | | Normal inhibition |
| B/Mozambique/IR981/2015 | 08/06/2015 | B(Yam) | 74.12 | | Normal inhibition | 13.03 | | Normal inhibition |
| B/Mozambique/IR1062/2015 | 23/06/2015 | B(Yam) | 29.81 | | Normal inhibition | 2.82 | | Normal inhibition |
| B/Mozambique/IR1010/2015 | 11/06/2015 | B(Yam) | 18.44 | | Normal inhibition | 4.15 | | Normal inhibition |

IC50 – the concentration of NA inhibitor which reduces NA activity by 50% of the virus.
