# Supplementary material for: Antigenic and genetic characterization of influenza viruses isolated in Mozambique during the 2015 season
Source: PLoS One. 2018 Jul 26;13(7):e0201248. doi: 10.1371/journal.pone.0201248 (PMC6062064; doi:10.1371/journal.pone.0201248)
Supplement: S2 Table — (DOC) [file pone.0201248.s004.doc]

| *HA sequences* | *Genetic group* | *Amino acid substitutions* | | | | | | | | | | | | | | | | | | |
| --- | --- | --- | --- | --- | --- | --- | --- | --- | --- | --- | --- | --- | --- | --- | --- | --- | --- | --- | --- | --- |
|  |  | 048 | 069 | 084 | 097 | 120 | 129 | 163 | 185 | 203 | 208 | 234 | 235 | 256 | 283 | 374 | 451 | 491 | 494 | 499 |
| ***A/California/7/2009*** |  | ***A*** | ***S*** | ***S*** | ***D*** | ***T*** | ***N*** | ***K*** | ***S*** | ***S*** | ***K*** | ***V*** | ***E*** | ***A*** | ***K*** | ***E*** | ***S*** | ***E*** | ***K*** | ***E*** |
| *A/Bayern/69/2009* |  | *.* | *.* | *.* | *.* | *.* | *.* | *.* | *.* | *.* | *.* | *.* | *.* | *.* | *.* | *.* | *.* | *.* | *.* | *.* |
| *A/Lviv/N6/2009* |  | *.* | *.* | *.* | *.* | *.* | *.* | *.* | *.* | *T* | *.* | *.* | *.* | *.* | *.* | *.* | *.* | *.* | *.* | *.* |
| *A/Christchurch/16/2010* | 4 | *.* | *.* | *.* | *.* | *.* | *.* | *.* | *.* | *T* | *.* | *.* | *.* | *.* | *.* | *K* | *.* | *.* | *.* | *.* |
| *A/Astrakhan/1/2011* | 5 | *.* | *.* | *.* | *.* | *.* | *.* | *.* | *.* | *T* | *.* | *.* | *.* | *.* | *.* | *K* | *.* | *.* | *.* | *.* |
| *A/St Petersburg/100/2011* | 6 | *.* | *.* | *.* | *.* | *.* | *.* | *.* | *T* | *T* | *.* | *.* | *.* | *.* | *.* | *K* | *N* | *.* | *.* | *.* |
| *A/St Petersburg/27/2011* | 7 | *.* | *.* | *.* | *N* | *.* | *.* | *.* | *T* | *T* | *.* | *.* | *.* | *.* | *.* | *K* | *N* | *.* | *.* | *.* |
| *A/Hong Kong/5659/2012* | 6A | *.* | *.* | *.* | *N* | *.* | *.* | *.* | *T* | *T* | *.* | *.* | *.* | *.* | *.* | *K* | *N* | *.* | *.* | *.* |
| *A/South Africa/3626/2013* | 6B | *.* | *.* | *.* | *N* | *.* | *.* | *Q* | *T* | *T* | *.* | *.* | *.* | *T* | *E* | *K* | *N* | *.* | *.* | *K* |
| A/Ghana/DILI-14-0620/2014 | 6B | . | . | . | N | . | . | . | T | T | . | I | . | . | E | K | N | . | . | K |
| A/Dakar/03/2014 | 6B | . | . | . | N | . | . | . | T | T | . | I | . | . | E | K | N | . | . | K |
| A/Norway/2227/2015 | 6B | . | . | . | N | . | . | Q | T | T | . | . | . | T | E | K | N | . | . | K |
| A/Iceland/39/2015 | 6B | . | . | . | N | . | . | Q | T | T | . | . | . | T | E | K | N | G | . | K |
| A/Jordan/11494/2015 | 6B | . | . | . | N | . | . | Q | T | T | . | . | . | T | E | K | N | G | . | K |
| A/Bangladesh/4005/2015 | 6B | . | . | . | N | . | D | Q | T | T | . | . | . | T | E | K | N | G | . | K |
| A/Paris/1686/2015 | 6B | . | . | . | N | . | . | Q | T | T | . | . | . | T | E | K | N | . | . | K |
| A/Peru/06/2015 | 6B | . | . | . | N | . | . | Q | T | T | . | . | . | T | E | K | N | . | . | K |
| A/St Petersburg/61/2015 | 6B | . | . | . | N | . | . | Q | T | T | . | . | . | T | E | K | N | . | . | K |
| A/Picardie/1479/2015 | 6B | . | . | . | N | . | . | Q | T | T | . | . | . | T | E | K | N | . | . | K |
| A/Norway/2330/2015 | 6B | . | . | N | N | . | . | Q | T | T | . | . | . | T | E | K | N | . | . | K |
| A/Zambia/06-00043/2015 | 6B | . | . | N | N | . | . | Q | T | T | . | . | . | T | E | K | N | . | . | K |
| A/Zambia/01-00118/2015 | 6B | . | . | N | N | . | . | Q | T | T | . | . | . | T | E | K | N | . | . | K |
| A/Zambia/01-00096/2015 | 6B | . | . | N | N | . | . | Q | T | T | . | . | . | T | E | K | N | . | . | K |
| A/Jordan/11917/2015 | 6B | . | . | N | N | . | . | Q | T | T | . | . | . | T | E | K | N | . | . | K |
| A/Jordan/20421/2015 | 6B | . | . | N | N | . | . | Q | T | T | . | . | . | T | E | K | N | . | R | K |
| A/Mozambique/IR495/2015 | 6B | . | P |  | N | A | . | Q | T | T | R | . | D | T | E | K | N | . | . | K |
| A/Mozambique/IR418/2015 | 6B | . | P |  | N | A | . | Q | T | T | R | . | D | T | E | K | N | . | . | K |
| A/Mozambique/IR543/2015 | 6B | . | P |  | N | A | . | Q | T | T | R | . | D | T | E | K | N | . | . | K |
| A/Mozambique/IR467/2015 | 6B | . | P |  | N | A | . | Q | T | T | R | . | D | T | E | K | N | . | . | K |

A – Alanine; D – Aspartate; E – Glutamate; G – Glicine; H – Histidine; I – Isoleucine; K – Lysine; N – Asparagine; P – Proline; Q – Glutamine; R – Arginine; S – Serine; T – Treonine; V – Valina.

Amino acid substitutions (specific symbol) are indicated for each HA sequence at correspondent position in comparison to A/California/7/2009; reference viruses used for antigenic analysis are indicated (italic) and amino acid substitutions observed only in Mozambique viruses (normal blue).
