# Supplementary material for: Antigenic and genetic characterization of influenza viruses isolated in Mozambique during the 2015 season
Source: PLoS One. 2018 Jul 26;13(7):e0201248. doi: 10.1371/journal.pone.0201248 (PMC6062064; doi:10.1371/journal.pone.0201248)
Supplement: S3 Table — (DOC) [file pone.0201248.s005.doc]

| *HA sequences* | *Genetic group* | *Amino acid substitutions* | | | | | | | | | | | | | | | | | | | | |
| --- | --- | --- | --- | --- | --- | --- | --- | --- | --- | --- | --- | --- | --- | --- | --- | --- | --- | --- | --- | --- | --- | --- |
|  |  | 3 | 3  3 | 4  5 | 6  2 | 1  2  2 | 1  2  4 | 1  4  4 | 1 4 5 | 1  5  9 | 1 6 0 | 1  8  3 | 1  8  6 | 1 9 4 | 1  9  7 | 2  1  2 | 2 2 3 | 2  2  5 | 2  7  8 | 3  1  1 | 4 8 9 | 5 0 5 |
| ***A/Perth/16/2009*** |  | ***L*** | ***Q*** | ***S*** | ***E*** | ***N*** | ***S*** | ***K*** | ***N*** | ***F*** | ***K*** | ***L*** | ***G*** | ***L*** | ***Q*** | ***T*** | ***V*** | ***N*** | ***N*** | ***Q*** | ***D*** | ***V*** |
| *A/Victoria/361/2011* |  | *.* | *R* | *N* | *K* | *.* | *.* | *N* | *.* | *.* | *.* | *H* | *.* | *.* | *.* | *A* | *I* | *.* | *K* | *.* | *.* | *.* |
| *A/Texas/50/2012* | *3C.1* | *.* | *R* | *N* | *K* | *.* | *.* | *N* | *.* | *.* | *.* | *H* | *.* | *.* | *.* | *A* | *I* | *.* | *K* | *.* | *.* | *.* |
| *A/Hong Kong/146/2013* | *3C.2* | *.* | *R* | *N* | *K* | *.* | *.* | *N* | *S* | *.* | *.* | *H* | *V* | *.* | *.* | *A* | *I* | *.* | *K* | *.* | *N* | *.* |
| *A/Hong Kong/4801/2014* | *3C.2* | *I* | *R* | *N* | *K* | *.* | *.* | *S* | *S* | *Y* | *.* | *H* | *V* | *P* | *.* | *A* | *I* | *D* | *K* | *H* | *N* | *.* |
| *A/Netherlands/525/2014* | *3C.2* | *.* | *R* | *N* | *K* | *.* | *.* | *N* | *S* | *.* | *.* | *H* | *V* | *.* | *.* | *A* | *I* | *.* | *K* | *.* | *.* | *.* |
| *A/Hong Kong/5738/2014* | *3C.2a* | *I* | *R* | *N* | *K* | *.* | *.* | *S* | *S* | *Y* | *.* | *H* | *V* | *P* | *.* | *A* | *I* | *D* | *K* | *H* | *N* | *.* |
| *A/Samara/73/2013* | *3C.3* | *.* | *R* | *N* | *K* | *.* | *.* | *N* | *S* | *.* | *.* | *H* | *V* |  | *.* | *A* | *I* | *.* | *K* | *.* | *.* | *.* |
| *A/Switzerland/9715293/2013* | *3C.3a* | *.* | *R* | *N* | *K* | *.* | *.* | *N* | *S* | *.* | *.* | *H* | *.* | *.* | *.* | *A* | *I* | *.* | *K* | *.* | *.* | *.* |
| *A/Stockholm/6/2014* | *3C.3a* | *.* | *R* | *N* | *K* | *.* | *.* | *N* | *S* | *.* | *.* | *H* | *.* | *.* | *.* | *A* | *I* | *.* | *K* | *.* | *.* | *.* |
| *A/Netherlands/525/2014* | 3C.3b | . | R | N | . | D | . | N | S | . | . | H | V | . | H | A | I | . | K | . | . | . |
| A/South Africa/R3989/2015 | 3C.2a | I | R | N | K | . | . | S | S | Y | T | H | V | . | R | A | I | D | K | H | N | . |
| A/South Africa/R3778/2015 | 3C.2a | I | R | N | K | . | . | S | S | Y | T | H | V | . | R | A | I | D | K | H | N | . |
| A/South Africa/R1734/2015 | 3C.2a | I | R | N | K | . | . | S | S | Y | T | H | V | . | R | A | I | D | K | H | N | . |
| A/South Africa/R3457/2015 | 3C.2a | I | R | N | K | . | . | S | S | Y | T | H | V | . | R | A | I | D | K | H | N | . |
| A/South Africa/R2825/2015 | 3C.2a | I | R | N | K | . | . | S | S | Y | T | H | V | . | R | A | I | D | K | H | N | . |
| A/South Africa/R3944/2015 | 3C.2a | I | R | N | K | . | . | S | S | Y | T | H | V | . | R | A | I | D | K | H | N | . |
| A/South Africa/R2490/2015 | 3C.2a | I | R | N | K | . | . | S | S | Y | T | H | V | . | R | A | I | D | K | H | N | . |
| A/Istanbul/1367/2015 | 3C.2a | I | R | N | K | . | . | S | S | Y | T | H | V | . | R | A | I | D | K | H | N | . |
| A/Mozambique/IR421/2015 | 3C.2a | I | R | N | K | . | . | S | S | Y | . | H | V | . | R | A | I | D | K | H | N | . |
| A/Mozambique/IR422/2015 | 3C.2a | I | R | N | K | . | R | S | S | Y | . | H | V | . | R | A | I | D | K | H | N | I |
| A/Mozambique/IR424/2015 | 3C.2a | I | R | N | K | . | . | S | S | Y | . | H | V | . | R | A | I | D | K | H | N | . |
| A/Mozambique/IR436/2015 | 3C.2a | I | R | N | K | . | . | S | S | Y | T | H | V | . | R | A | I | D | K | H | N | . |
| A/Mozambique/IR451/2015 | 3C.2a | I | R | N | K | . | . | S | S | Y | . | H | V | . | R | A | I | D | K | H | N | . |
| A/Mozambique/IR454/2015 | 3C.2a | I | R | N | K | . | R | S | S | Y | . | H | V | . | R | A | I | D | K | H | N | I |
| A/Mozambique/IR479/2015 | 3C.2a | I | R | N | K | . | . | S | S | Y | . | H | V | . | R | A | I | D | K | H | N | . |
| A/Mozambique/IR481/2015 | 3C.2a | I | R | N | K | . | . | S | S | Y | . | H | V | . | R | A | I | D | K | H | N | . |
| A/Mozambique/IR493/2015 | 3C.2a | I | R | N | K | . | . | S | S | Y | T | H | V | . | R | A | I | D | K | H | N | . |
| A/Mozambique/IR499/2015 | 3C.2a | I | R | N | K | . | . | S | S | Y | . | H | V | . | R | A | I | D | K | H | N | . |
| A/Mozambique/IR538/2015 | 3C.2a | I | R | N | K | . | . | S | S | Y | . | H | V | . | R | A | I | D | K | H | N | . |
| A/Mozambique/IR803/2015 | 3C.2a | I | R | N | K | . | . | S | S | Y | T | H | V | . | R | A | I | D | K | H | N | . |

A – Alanine; D – Aspartate; E – Glutamate; G – Glicine; H – Histidine; I – Isoleucine; K – Lysine; L – Leucine; N – Asparagine; Q – Glutamine; R – Arginine; S – Serine; T – Treonine; V – Valina; Y – Tyrosine.

Amino acid substitutions (specific symbol) are indicated for each HA sequence at correspondent position in comparison to A/Perth/16/2009; reference viruses used for antigenic analysis are indicated (italic) and amino acid substitutions observed in Mozambique viruses and other genetically and/or antigenically similar viruses (normal blue).
