# Supplementary material for: Antigenic and genetic characterization of influenza viruses isolated in Mozambique during the 2015 season
Source: PLoS One. 2018 Jul 26;13(7):e0201248. doi: 10.1371/journal.pone.0201248 (PMC6062064; doi:10.1371/journal.pone.0201248)
Supplement: S4 Table — (DOC) [file pone.0201248.s006.doc]

| *HA sequences* | *Genetic group* | *Amino acid substitutions* | | | | | | | | | | | | | | | |
| --- | --- | --- | --- | --- | --- | --- | --- | --- | --- | --- | --- | --- | --- | --- | --- | --- | --- |
|  |  | 048 | 108 | 116 | 1  25 | 150 | 165 | 172 | 181 | 196 | 202 | 229 | 2  32 | 251 | 298 | 312 | 5  04 |
| ***B/Florida/4/2006*** | ***1*** | ***R*** | ***P*** | ***N*** | ***I*** | ***S*** | ***N*** | ***L*** | ***T*** | ***D*** | ***N*** | ***G*** | ***D*** | ***M*** | ***K*** | ***E*** | ***N*** |
| *B/Brisbane/3/2007* | *2* | *K* | *A* | *.* | *.* | *.* | *.* | *.* | *.* | *.* | *.* | *.* | *.* | *.* | *.* | *.* | *.* |
| *B/Estonia/55669/2011* | *2* | *K* | *A* | *.* | *.* | *.* | *.* | *.* | *A* | *N* | *.* | *.* | *.* | *.* | *.* | *.* | *.* |
| *B/Massachusetts/02/2012* | *2* | *K* | *A* | *.* | *.* | *.* | *.* | *.* | *A* | *.* | *.* | *.* | *.* | *.* | *.* | *.* | *.* |
| *B/Wisconsin/1/2010* | *3* | *.* | *.* | *.* | *.* | *I* | *Y* | *.* | *.* | *N* | *S* | *D* | *.* | *.* | *.* | *.* | *.* |
| *B/Stockholm/12/2011* | *3* | *.* | *.* | *.* | *.* | *I* | *Y* | *.* | *.* | *.* | *.* | *D* | *.* | *.* | *.* | *.* | *.* |
| *B/Phuket/3073/2013* | *3* | *.* | *.* | *K* | *.* | *I* | *Y* | *.* | *.* | *N* | *S* | *D* | *.* | *.* | *E* | *K* | *.* |
| *B/Hong Kong/3417/2014* | *3* | *.* | *.* | *K* | *.* | *I* | *Y* | *Q* | *.* | *N* | *S* | *D* | *.* | *.* | *E* | *K* | *.* |
| B/Dakar/08/2015 | 3 | . | . | K | . | I | Y | . | . | N | S | D | . | V | E | K | . |
| B/Ghana/DILI-15-0295/2015 | 3 | . | . | K | . | I | Y | . | . | N | S | D | . | V | E | K | . |
| B/Antananarivo/1315/2015 | 3 | . | . | K | . | I | Y | Q | . | N | S | D | . | V | E | K | . |
| B/Zambia/04-00173/2015 | 3 | . | . | K | . | I | Y | . | . | N | S | D | . | V | E | K | . |
| B/South Africa/R3192/2015 | 3 | . | . | K | L | I | Y | . | . | N | S | D | N | V | E | K | D |
| B/South Africa/R3183/2015 | 3 | . | . | K | L | I | Y | . | . | N | S | D | N | V | E | K | D |
| B/South Africa/R3762/2015 | 3 | . | . | K | L | I | Y | . | . | N | S | D | N | V | E | K | D |
| B/Mozambique/IR981/2015 | 3 | . | . | K | L | I | Y | . | . | N | S | D | N | V | E | K | D |
| B/Mozambique/IR1010/2015 | 3 | . | . | K | L | I | Y | . | . | N | S | D | N | V | E | K | D |
| B/Mozambique/IR1062/2015 | 3 | . | . | K | L | I | Y | . | . | N | S | D | N | V | E | K | D |

A – Alanine; D – Aspartate; E – Glutamate; G – Glicine; H – Histidine; I – Isoleucine; K – Lysine;

L – Leucine; M – Methionine; N – Asparagine; P – Proline; Q – Glutamine; R – Arginine; S – Serine; T – Treonine; V – Valina; Y – Tyrosine.

Amino acid substitutions (specific symbol) are indicated for each HA sequence at correspondent position in comparison to B/Florida/4/2006; reference viruses used for antigenic analysis are indicated (italic) and amino acid substitutions observed only in Mozambique viruses (normal blue).
